# Allometric relationships of ecologically important Antarctic and Arctic zooplankton and fish species

**DOI:** 10.1007/s00300-021-02984-4

**Published:** 2022-01-08

**Authors:** Fokje L. Schaafsma, Carmen L. David, Doreen Kohlbach, Julia Ehrlich, Giulia Castellani, Benjamin A. Lange, Martina Vortkamp, André Meijboom, Anna Fortuna-Wünsch, Antonia Immerz, Hannelore Cantzler, Apasiri Klasmeier, Nadezhda Zakharova, Katrin Schmidt, Anton P. Van de Putte, Jan Andries van Franeker, Hauke Flores

**Affiliations:** 1 Wageningen Marine Research, Ankerpark 27, 1871 AG Den Helder, The Netherlands; 2 Alfred-Wegener-Institute Helmholtz-Centre for Polar- and Marine Research, Bremerhaven, Germany; 3 Department of Biology, Dalhousie University, Halifax, B3K 4R2 Canada; 4 Biology Department, Woods Hole Oceanographic Institution, Woods Hole, Falmouth, MA 02543 USA; 5 Fram Centre, Norwegian Polar Institute, 9296 Tromsø, Norway; 6 Centre for Natural History (CeNak), University of Hamburg, Martin‑Luther‑King‑Platz 3, 20146 Hamburg, Germany; 7 Department of Biology, Ruhr University Bochum, 44801 Bochum, Germany; 8 Faculty of Mathematics, Informatics and Natural Sciences, University of Hamburg, Welckerstraße 8, 20148 Hamburg, Germany; 9 Institute of Earth Sciences, St.Petersburg State University, Universitetskaya Nab. 7-9, 199034 St. Petersburg, Russia; 10 School of Geography, Earth and Environmental Sciences, University of Plymouth, Plymouth, UK; 11 Royal Belgian Institute of Natural Sciences, Vautierstraat 29, B-1000 Brussels, Belgium; 12 Université Libre de Bruxelles, 50, Avenue FD Roosevelt, B-1050 Brussels, Belgium

**Keywords:** Arctic Ocean, Southern Ocean, Length, Mass, Zooplankton, Fish, Regression models

## Abstract

Allometric relationships between body properties of animals are useful for a wide variety of purposes, such as estimation of biomass, growth, population structure, bioenergetic modelling and carbon flux studies. This study summarizes allometric relationships of zooplankton and nekton species that play major roles in polar marine food webs. Measurements were performed on 639 individuals of 15 species sampled during three expeditions in the Southern Ocean (winter and summer) and 2374 individuals of 14 species sampled during three expeditions in the Arctic Ocean (spring and summer). The information provided by this study fills current knowledge gaps on relationships between length and wet/dry mass of understudied animals, such as various gelatinous zooplankton, and of animals from understudied seasons and maturity stages, for example, for the krill *Thysanoessa macrura* and larval *Euphausia superba* caught in winter. Comparisons show that there is intra-specific variation in length–mass relationships of several species depending on season, e.g. for the amphipod *Themisto libellula*. To investigate the potential use of generalized regression models, comparisons between sexes, maturity stages or age classes were performed and are discussed, such as for the several krill species and *T. libellula*. Regression model comparisons on age classes of the fish *E. antarctica* were inconclusive about their general use. Other allometric measurements performed on carapaces, eyes, heads, telsons, tails and otoliths provided models that proved to be useful for estimating length or mass in, e.g. diet studies. In some cases, the suitability of these models may depend on species or developmental stages.

**Supplementary Information:**

The online version contains supplementary material available at 10.1007/s00300-021-02984-4.

## Introduction

Measurements on size, mass and other properties of species are of fundamental interest in biology and important for the use in ecosystem studies and biogeochemical models. Allometric relationships derived from these measurements can be used for studying a variety of biological and ecological principles, such as morphology, predator–prey relationships, food web structure and the environmental impact on certain body parameters (Froese 2006). They can also be used for the comparison of, e.g. growth, energy expenditure, population structure and body condition between groups of individuals (Froese 2006). The direct measurement of many body parameters is often complicated due to, for example, time or logistical constraints. Therefore, regression models of allometric relationships can be useful to fill data gaps or reduce the need for time-consuming measurements. In addition, such relationships are often used to estimate biomass and production derived from length/frequency data in hydroacoustic surveys (Siegel 1992; Geoffroy et al. 2016) and to estimate biomass when using tools that do not allow for direct measurements. Examples of such tools are the Lightframe On-sight Keyspecies Investigation (LOKI; Schulz et al. 2010) system or ZooScan (Gorsky et al. 2010), which are image-based tools that are increasingly used for semiautomatically obtaining data on species identification and enumeration. Furthermore, diet studies often rely on body parts to identify food items and reconstruct an estimated biomass of the ingested food (Van Franeker et al. 2001; Fijn et al. 2012; Leopold et al. 2015; Schaafsma et al. 2017). Regressions of relationships between, e.g. length and the size of a certain body part are essential for these purposes.

Seasonal, regional and/or annual variation in individual mass per given length is found for many species (e.g. Nicol et al. 2000; Froese 2006; Dubishar et al. 2012) due to, for example, timing of life cycle events or variability in food availability. Furthermore, the individual mass of a species can vary for a certain length depending on developmental stage due to differences in morphological characteristics. Such differences in morphological parameters may have a large impact on biomass estimates (e.g. Ashjian et al 2003). A lack of knowledge on possible sources of variation makes it hard to assess the accuracy of regression models used and, thus, of the outcome of the analysis. In addition, a lack of information about the length–mass relationships of a species in certain seasons or for certain ontogenetic stages can hamper the ability to obtain accurate biomass estimates for modelling of food webs and studying ecosystems (Kulbicki et al 2005; Saunders et al. 2020). The meta-analysis of a large number of regression models can provide important insights into the ecology of well-studied species (Froese 2006; Ogle 2016) and the careful inspection of length–mass regressions of individuals of a species collected at different times and places can indicate if generalizations can be made for different ages, season or regions (Culver et al. 1985). Especially in the polar regions, data collections are often temporally and spatially limited, warranting the need for the publication of allometric data for a general use (Xavier et al. 2020). The availability of data from these regions and a concomitant assessment of these data would aid governance and conservation of polar ecosystems and management of current and future fisheries, particularly as the polar regions are very vulnerable to climate change (Reid 2018).

In the framework of the Dutch and German *Iceflux* projects, which studied the importance of sea ice in supporting polar marine living resources and the contribution of sea-ice-derived carbon to the carbon flux within polar ecosystems, allometric measurements were performed on several Antarctic and Arctic marine animals. These measurements were necessary for studies on, for example, population dynamics (Schaafsma et al. 2016), community structure (David et al. 2015; Ehrlich et al. 2020), biomass and carbon flux (David et al. 2017; Flores et al. 2019), energy content (Schaafsma et al. 2018), food web dynamics and cryo-pelagic carbon flux (Ehrlich et al. 2021; Kohlbach et al. 2016; 2017a, b, 2018, 2019; Schaafsma et al. 2017), calculating, for example, the biomass of zooplankton in a particular area, lipid content per dry mass or stomach contents per life stage. In many studies, the length measurements themselves are rarely made available as they are often of indirect interest and only used to estimate the other variables, such as aforementioned examples (Morris et al. 1988). This paper provides a detailed record of length, mass and other allometric measurements performed on zooplankton and nekton collected in the Antarctic and Arctic regions during six expeditions between 2012 and 2017. The aim of this study is to provide regression models of allometric relationships between several units of length and mass to the scientific community and to assess the variability of these regression models considering the season, geographical region and life cycle. In addition, regressions were performed between total length or mass and the size of body parts, such as carapace length (krill), telson length, head length and eye length (amphipods), otolith length (fish), tail length and head width (chaetognaths).

## Materials and methods

### Sample collection

**Fig. 1:**
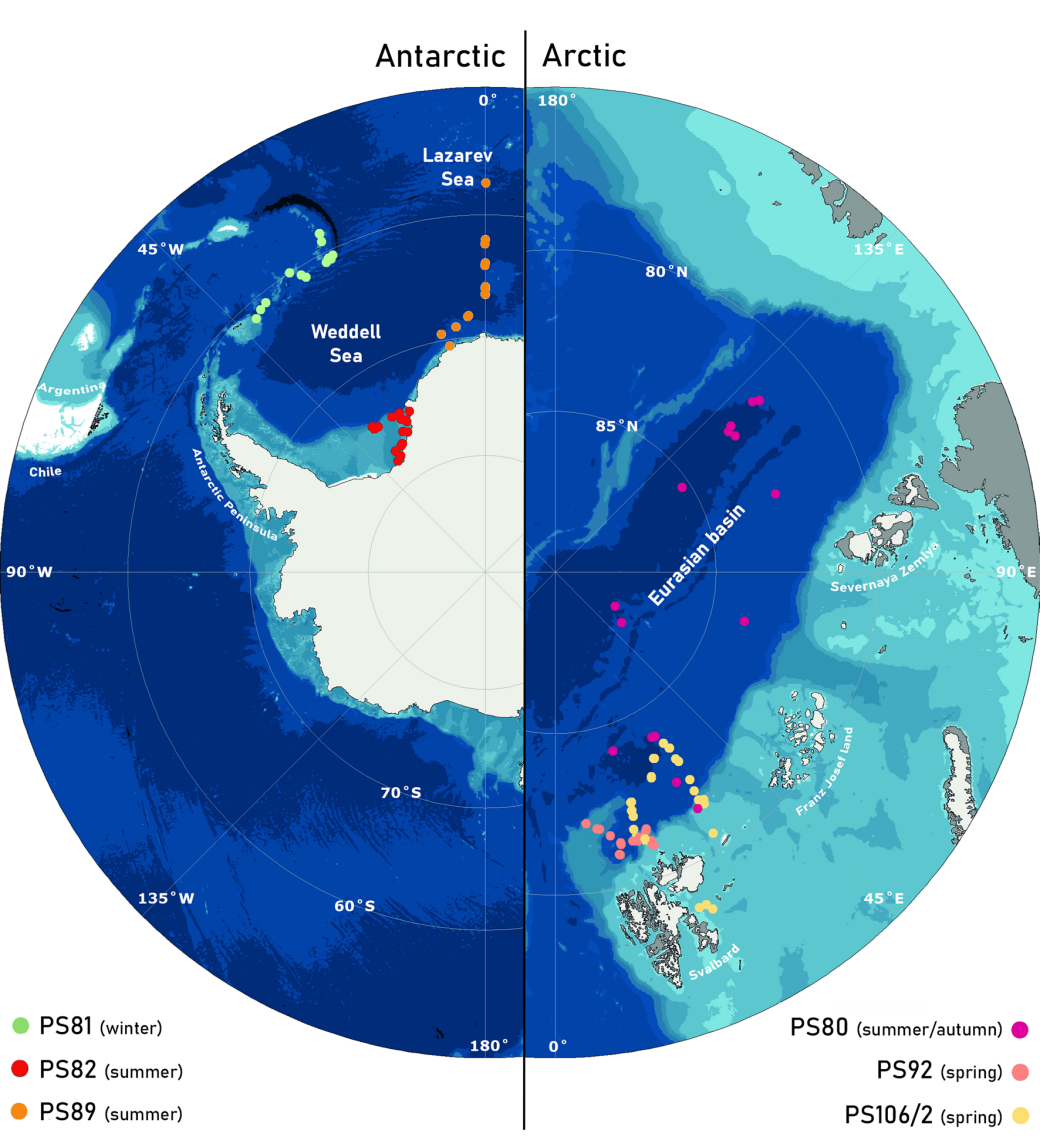
Maps of sampling locations (coloured dots) in the Southern and Arctic Oceans indicated by their *Polarstern* expedition number. Expeditions were conducted in August–October 2012 (PS80), May–June 2015 (PS92), June–July 2017 (PS106/2), August–October 2013 (PS81), December–March 2013/2014 (PS82) and December–February 2014/2015 (PS89). Seasons refer to austral seasons in case of Antarctic expeditions

Animals were collected during three Antarctic and three Arctic expeditions on board RV *Polarstern* (Fig. 1; Table 1). The Antarctic expeditions took place in the northern Weddell Sea from August to October 2013 (PS81), off the Filchner–Ronne ice shelf from December 2013 to March 2014 (PS82) and in the Lazarev Sea from December 2014 to February 2015 (PS89). The Arctic expeditions took place in the Eurasian Basin of the Arctic Ocean from August to October 2012 (PS80) and north of Svalbard from May to June 2015 (PS92) and June to July 2017 (PS106/2). Samples were generally collected in the upper two metres of the water column (both open water and ice-covered water) with a Surface and Under-Ice Trawl (SUIT; van Franeker et al. 2009) or at deeper depth layers with a Rectangular Midwater Trawl (RMT). The SUIT had a steel frame with a 2 by 2 m net opening, with a 7-mm half-mesh commercial shrimp net over 1.5-m width and a 0.3-mm mesh plankton net over 0.5-m width. Floats attached to the top of the frame kept the net at the surface or directly under the ice. The SUIT sheared out to the side of the ship, sampling away from the ship’s wake and under relatively undisturbed sea ice (Van Franeker et al. 2009; Suppl. mat. in Flores et al. 2012). The RMT consisted of an RMT-1 with a 0.33-mm mesh mounted above an RMT-8 with a mesh size of 4.5 mm at the opening and 0.85 mm at the cod end. The net openings were 1 and 8 m^2^, respectively. The 0–500 m stratum and the 0–100 m stratum were sampled by the RMT during PS81 and PS106/2, respectively. During some expeditions (PS82, PS89 and PS92) an opening/closing multi-RMT was used with the same sized nets, sampling different depth strata (usually either 0–50 m, 50–100 m and 100–200 m or 0–50 m, 50–200 m and 200–500 or 0–200 m, 200–500 m and 500–1000 m, depending on bottom depth). During PS106/2 some samples were obtained by a multinet (MN) or a bottom trawl (BT). The MN sampled at depths between 100 and 1500 m, whilst the BT sampled at depths between 200 and 800 m. During all expeditions, samples were collected in areas that were completely or partially ice-covered, except for the BT samples that were collected in an area were the ice had just retreated. More details of sampling and the sampled areas can be found in the respective cruise reports (PS80: Boetius 2013; PS81: Meyer and Auerwald 2014; PS82: Knust and Schröder 2014; PS89: Boebel 2015; PS92 Peeken 2016; PS106/2: Macke and Flores 2018). Environmental conditions were measured in the surface waters during trawling using several sensors mounted in the SUIT net frame, including temperature, salinity, chlorophyll *a* (in water and ice) and sea-ice thickness. These environmental conditions can be found in Lange et al. (2016, 2017) and are summarized in Castellani et al. (2020) for all expeditions.

**Table 1:**
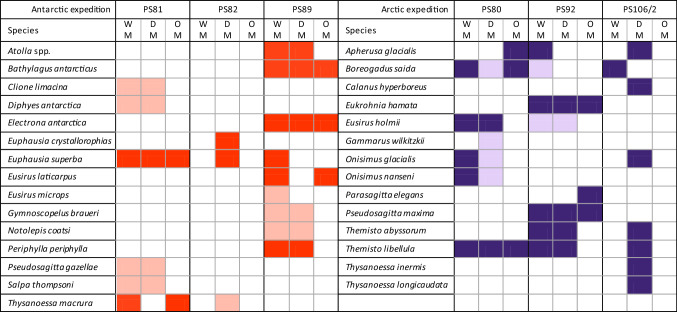
Overview of collected data per Antarctic (red, left) and Arctic (purple, right) expedition

Total lengths were recorded for all species except the cnidarians *Atolla* spp., *Diphyes antarctica* and *Periphylla periphylla*. For all fish except *Notolepis coatsi* standard length was also measured. Dark coloured squares indicate data that is further used for modelling. Light coloured squares indicate that there is data available but *n* is too small (< 10) for further analysis. These latter measurements are not further discussed in the text but can be found in Online Resources 1 and 2. WM = wet mass, DM = dry mass and OM = other measurements

### Measurements

Table 1 provides an overview of the species included in this study. All measurements were conducted on preserved specimens, except for *Euphausia superba* (Antarctic krill),* Euphausia crystallorophias* (Ice krill) and *Thysanoessa macrura* collected during PS82, which were measured before preservation. The length and wet mass (WM) of frozen individuals were measured after thawing. Dry mass (DM) was measured after animals were freeze-dried until complete desiccation. Masses were measured with a precision between a tenth and a thousandth of a milligram, depending on the size of the organism and the purpose of the measurement. All allometric measurements can be found in Online Resource 1 (Antarctic) and Online Resource 2 (Arctic) in which Darwin Core standard terms (Wieczorek et al. 2012) were used. These tables include information on the time and location of sample collection, which gear was used to catch the animals and the preservation method (4% buffered formaldehyde, or frozen at − 20 °C or − 80 °C).

Preservation can have an influence on mass and size. Fixation in a formalin solution can cause a loss in WM (Wetzel et al. 2005) and shrinkage (Wallis et al. 2020). For example, Wallis et al. (2020) estimated the total shrinkage of the krill *T. macrura* to be 1.4% after 1.5 years in a 10% formaldehyde solution. In addition, it has been shown that both formalin fixation and freezing can cause loss in DM (Williams and Robins 1982). The occurrence and extent of the loss, however, depends on the species, the size and developmental stage, and the amount of time the specimen was preserved (Williams and Robins 1982; Ogle 2009 and references therein; Wallis et al. 2020). Therefore, Online Resources 1 and 2 do not only include information on when the samples were caught, but also on when the sampled individuals were processed.

**Fig. 2:**
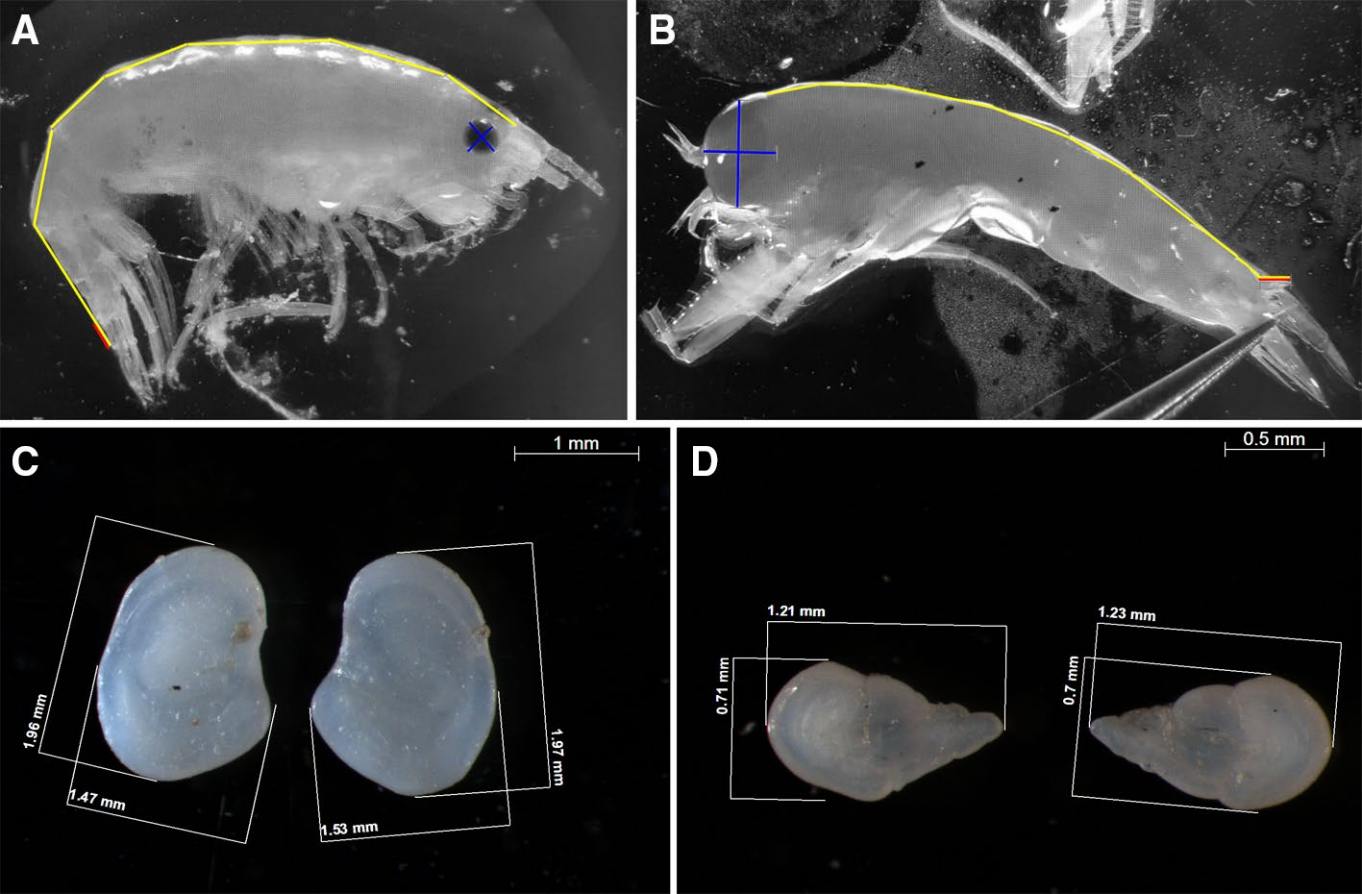
Examples of measurements on the gammarid amphipod *Apherusa glacialis* (**A**) and the hyperiid amphipod *Themisto libellula* (**B**), including total length (yellow line), horizontal and vertical eye lengths (blue lines) and telson length (red line), and of measurements on the otoliths of the fishes *Electrona antarctica* (**C**) and *Bathylagus antarcticus* (**D**)

The total length (TL) of different taxonomic groups was measured according to the most conventional methods used in scientific literature, aiming to ensure the possibility of comparison between studies. Details of each measurement are listed in Table 2. The TL of krill species is often measured from the tip of the rostrum to the tip of the telson, which was also done here for the species *T. macrura*, *E. crystallorophias*, *Thysanoessa inermis* and *Thysanoessa longicaudata*. The TL of *E. superba* was, however, measured to the nearest mm below from the anterior margin of the eye to the tip of the telson according to the widely used ‘Discovery method’ (Marr 1962). The TL of various suborders of amphipods are also measured slightly differently. Gammarid amphipods were measured following the curved dorsal line from the tip of the rostrum to the tip of the telson (Chapelle and Peck 2004; Krapp et al. 2008; Fig. 2A). Hyperiid amphipods were measured following the curved dorsal line from the front of the head to the tip of the telson (Pakhomov and Perissinotto 1996; Donnelly et al. 2006; Fig. 2B). For fish, the standard length (SL) was measured in addition to the total length (Table 2).

**Table 2:** Length parameters and their abbreviations used in this study

Parameter	Acronym	Taxonomic group	Description
Carapace length	CL	Krill	From the tip of the rostrum to the mid-dorsal posterior edge of the carapace
Eye distance	ED	Chaetognaths	Distance measured between the centers of both eyes
Eye length horizontal	ELH	Amphipods	Maximum width of the eye
Eye length vertical	ELV	Amphipods	Maximum height of the eye
Head length	HL	Amphipods	Length of the cephalon, measured from the tip of the rostrum
Head width	HW	Chaetognaths	Measured at the broadest part of the head
Otolith length	OL	Fish	Maximum length of the otolith
Otolith width	OW	Fish	Maximum width of the otolith
Prosome length	PL	Copepods	Measured from the tip of the cephalosome to the distal lateral end of the last thoracic segment
Standard length	SL	Fish	Measured from the tip of the snout to the end of the last vertebra
Total length	TL	1. Krill (*Euphausia superba*) 2. Krill (other species) 3. Gammarid amphipods 4. Hyperiid amphipods 5. Fish 6. Chaetognaths	1. Measured from the anterior margin of the eye to the tip of the telson 2. Measured from the tip of the rostrum to the tip of the telson 3. Measured following the curved dorsal line from the tip of the rostrum to the tip of the telson 4. Measured following the curved dorsal line from the front of the head to the tip of the telson 5. Measured from the tip of the snout to the posterior margin of the caudal fin 6. Measured from the front of the head to the tip of the tail excluding the tail fin
Tail length	TLL	Chaetognaths	Measured to the tip excluding the tail fin
Telson length	TSL	Amphipods	Length of the telson, measured dorsally

Other length measurements performed (Table 2) include head length (HL) for the Antarctic amphipod *Eusirus laticarpus*, eye length (horizontal ELH and vertical ELV) and telson length (TSL) for the Arctic amphipod species *Apherusa glacialis* and *Themisto libellula*, (as shown in Fig. 2A and B), carapace length (CL) of the krill *E. superba* and *T. macrura*, prosome length (PL) for the copepod *Calanus hyperboreus*, eye distance (ED), head width (HW) and tail length (TLL) for the chaetognaths *Eukrohnia hamata* and *Parasagitta elegans*, otolith length (OL) for the fish species *Boreogadus saida* (polar cod) and otolith length and width (OW) for the fishes *Electrona antarctica* and *Bathylagus antarcticus* (examples in Fig. 2C and D). For the calculation of allometric relationships between otolith parameters and measurements of body size, an average value of the left and right otolith per individual was used, as no difference was found between regression models using one or the other in a previous study performed on Southern Ocean myctophid fish (Saunders et al. 2020). Sexual maturity of *E. superba* collected during PS81 and PS89 was assessed according to Kirkwood (1982) and Makarov and Denys (1981). Due to the nature of the intended analyses, detailed information on sexual maturity was not necessary for the Antarctic krill collected during PS82, which were, therefore, sorted into juveniles, males and females according to CCAMLR (2011). The individuals of the fish species *E. antarctica* were divided in age classes using the length-at-age regression (Van de Putte et al. 2006) adapted from the growth curve equation from Greely et al. (1999).

### Statistics

Linear relationships between a length parameter (Table 2) and WM or DM were established on log_10_-transformed data according to

1 $$log_{10} \left( M \right) = log_{10} \left( a \right) + b log_{10} \left( L \right)$$

corresponding to the power function

2 $$M = a L^{b}$$

where *M* is mass (WM or DM) and *L* is a length parameter (Table 2). Regression constants *a* and *b* were estimated using least squares regression.

Linear relationships between the different measurements of length or different measurements of mass were established as

3 $$y = ax + b$$

where *y* is the response variable and *x* the explanatory variable.

All regression models were plotted and individuals with masses that were double or half the expected mass based on the model were considered outliers and removed (Jellyman et al. 2013). Eight measurements were removed in total (one of *E. hamata*, one of *E. superba* and six of *T. libellula*) and can be found in Online Resource 3. Given the values of these outliers, we suspect that these represent measurement errors. As length–mass relationships can be affected by life stage or age class, life stages with an expected difference in morphology or obviously different size/age classes with small numbers were excluded from the models as indicated in the text to avoid misleading results (Froese 2006; Jellyman et al. 2013).

To check the linear regression assumptions of the normal distribution and constant variance of measurement errors, residual plots and histograms of the model residuals were assessed visually (Ogle 2016). The coefficient of determination (*R*^2^), representing the proportion of the variance of a dependent variable that is explained by the independent variable of the regression model, was given as a measure of how well the linear model predicts the measured values. In order to investigate potential intra-specific differences in the regression models caused by, e.g. sex, developmental stage or season, the slopes and intercepts of the linear regressions were compared with ANCOVA (Hartman and Brandt 1995; Ogle 2016), using version 4.0.2 of R with the packages “car” (Fox and Weisberg 2019), “FSA” (Ogle et al. 2020) and “dplyr” (Wickham et al. 2020). Statistical significance was defined as *α* ≤ 0.05. The residual standard deviation is given when models from the same dataset were compared with ANCOVA, representing the average amount that the real measured values of *y* differed from the predictions provided by the regression line given in model comparisons. The R package “ggplot2” (Wickham 2016) was used for visualization.

## Results

### General total length–mass regressions

#### Krill

Measurements were performed on krill species (Euphausiidae) from both the Southern Ocean and the Arctic Ocean. For *E. superba*, TL and WM, and TL and DM were measured on specimens caught during austral summer (PS89 and PS82, respectively). The TL–DM model for krill from PS82 violated the homogenous variance assumption. This could be due to the wide range of stages that were present, including gravid females, possibly combined with a relatively small sample size (*n* = 37). TL, WM and DM were measured on specimens collected during austral winter (PS81). The TL–WM model given for austral winter (Table 3) was based on age-class 0 (AC0) Antarctic krill < 24 mm TL, consisting of furcilia larvae and juveniles (Schaafsma et al. 2016). The WM measurements of sub-adult and adult Antarctic krill from PS81 (*n* = 10) were excluded from the model. The TL–DM model for PS81 was based on sub-adult and adult Antarctic krill (Table 3). Due to morphological differences, age-class 0 krill (*n* = 7) were excluded from this model. All measurements are provided in Online Resource 1. A regression model between WM and DM for *E. superba* caught during winter can be found in Table 4.

**Table 3:** Overview of regression parameters and *R*^2^ of linear regression models on log_10_-transformed length and mass data (log_10_(*M*) = log_10_(*a*) + *b* log_10_(*L*)) and the corresponding power function (*M* = *a L*^*b*^) from a variety of Arctic and Antarctic species

Species	Season (Expedition)	Length range (TL) (mm)	*x*	*y*	*n*	log *a*	*a*	*b*	*R*^*2*^
Antarctic									
*Bathylagus antarcticus*	Summer (PS89)	34–58	TL (mm)	WM (mg)	11	− 6.718	0.000	3.797	0.980
*Bathylagus antarcticus*	Summer (PS89)	34–58	TL (mm)	DM (mg)	11	− 7.391	0.000	3.725	0.983
*Eusirus laticarpus*	Summer (PS89)	12.5–17.7	TL (mm)	WM (mg)	14	− 0.657	0.220	2.113	0.693
*Euphausia crystallorophias*^*c*^	Summer (PS82)	20–33	TL (mm)	DM (mg)	40	− 3.246	0.001	3.372	0.803
*Euphausia superba*	Winter (PS81)	7–22	TL (mm)	WM (mg)	109	− 2.318	0.005	3.060	0.904
*Euphausia superba*	Winter (PS81)	28–55	TL (mm)	DM (mg)	28	− 3.089	0.001	3.153	0.951
*Euphausia superba*^*c*^	Summer (PS89)	19–49	TL (mm)	WM (mg)	215	− 2.011	0.010	2.900	0.889
*Thysanoessa macrura*	Winter (PS81)	9–26	TL (mm)	WM (mg)	21	− 2.638	0.002	3.298	0.992
Arctic									
*Apherusa glacialis*	Spring (PS92)	7.6–12	TL (mm)	WM (mg)	27	− 1.364	0.043	2.544	0.737
*Apherusa glacialis*^*d*^	Spring (PS106/2)	8.4–15.5	TL (mm)	DM (mg)	97	− 2.180	0.007	2.731	0.760
*Boreogadus saida*	Spring (PS106/2)	86–177	TL (mm)	WM (g)	54	− 4.909	0.000	2.877	0.916
*Boreogadus saida*^*e*^	Sum/Autumn (PS80)	52–137	TL (mm)	WM (g)	119	− 5.239	0.000	3.018	0.966
*Eukrohnia hamata*	Spring (PS92)	20.0–31.9	TL (mm)	WM (mg)	25	− 3.222	0.001	3.403	0.821
*Eukrohnia hamata*^*f*^	Spring (PS92)	20.0–31.9	TL (mm)	DM (mg)	26	− 3.000	0.001	2.604	0.781
*Eusirus holmi*	Sum/Autumn (PS80)	30.6–37.4	TL (mm)	WM (mg)	37	− 1.116	0.077	2.438	0.466
*Eusirus holmi*	Sum/Autumn (PS80)	29.8–39.2	TL (mm)	DM (mg)	14	− 1.740	0.018	2.375	0.389
*Onisimus glacialis*	Sum/Autumn (PS80)	12.1–19.0	TL (mm)	WM (mg)	33	− 0.228	0.592	1.712	0.660
*Onisimus glacialis*	Spring (PS106/2)	10.9–14.6	TL (mm)	DM (mg)	14	− 1.457	0.035	2.070	0.460
*Onisimus nanseni*	Sum/Autumn (PS80)	11.6–23.9	TL (mm)	WM (mg)	11	− 1.412	0.039	2.658	0.963
*Themisto abyssorum*	Spring (PS92)	9.5–20.3	TL (mm)	WM (mg)	25	− 1.870	0.014	3.102	0.918
*Themisto abyssorum*	Spring (PS92)	9.5–20.3	TL (mm)	DM (mg)	25	− 1.559	0.028	2.143	0.837
*Themisto libellula*^*c*^	Sum/Autumn (PS80)	6–28	TL (mm)	WM (mg)	58	− 1.595	0.025	2.893	0.988
*Themisto libellula*	Sum/Autumn (PS80)	8.0–31.2	TL (mm)	DM (mg)	22	− 2.316	0.005	2.970	0.973
*Themisto libellula*	Spring (PS92)	15.3–40.2	TL (mm)	WM (mg)	60	− 2.420	0.004	3.304	0.918
*Themisto libellula*	Spring (PS92)	15.3–40.2	TL (mm)	DM (mg)	38	− 3.345	0.000	3.480	0.937
*Themisto libellula*	Spring (PS106/2)	3.7–34.1	TL (mm)	DM (mg)	81	− 2.102	0.008	2.969	0.943
*Thysanoessa inermis*	Spring (PS106/2)	18.2–31.2	TL (mm)	DM (mg)	44	− 2.721	0.002	2.969	0.498
*Thysanoessa longicaudata*	Spring (PS106/2)	8.2–18.9	TL (mm)	DM (mg)	67	− 3.523	0.0003	3.499	0.874

Length (*L*) = Total length (TL), Mass (*M*) = Wet mass (WM) or dry mass (DM). Seasons refer to austral seasons in case of Antarctic species

^c^Data was further analysed for differences between sexes/stages/ages. Results are presented in Table 5

^d^Previously published in Zakharova (2019)

^e^Previously published in David et al. (2016)

^f^Previously published in Immerz (2016)

**Table 4:** Overview of regression parameters and *R*^*2*^ of linear regression models (*y* = *ax* + *b*) between various measurements of length or mass from a variety of Arctic and Antarctic species

Species	Season (Expedition)	Length range (TL)	*x*	*y*	*n*	*a*	*b*	*R*^*2*^
Antarctic								
*Atolla* spp.	Summer (PS89)	NA	WM (g)	DM (g)	17	0.039	0.395	0.879
*Bathylagus antarcticus*	Summer (PS89)	34–58 mm	TL (mm)	SL (mm)	11	0.989	− 2.227	0.987
*Bathylagus antarcticus*	Summer (PS89)	34–58 mm	WM (g)	DM (g)	11	0.155	0.002	0.999
*Electrona antarctica*	Summer (PS89)	22–84 mm	TL (mm)	SL (mm)	68	0.921	− 0.295	0.996
*Electrona antarctica*	Summer (PS89)	22–84 mm	WM (g)	DM (g)	48	0.333	− 0.026	0.981
*Euphausia superba*	Winter (PS81)	10.3–36 mm	WM (mg)	DM (mg)	14	0.216	− 0.449	0.996
*Periphylla periphylla*	Summer (PS89)	NA	WM (g)	DM (g)	12	0.05	0.207	0.909
Arctic								
*Boreogadus saida*	Spring/Summer	86–182 mm	TL (mm)	SL (mm)	217	0.905	0.669	0.999
*Eukrohnia hamata*	Spring (PS92)	20.0–31.9 mm	WM (mg)	DM (mg)	25	0.092	1.355	0.817
*Themisto abyssorym*	Spring (PS92)	9.5–20.3 mm	WM (mg)	DM (mg)	25	0.095	3.155	0.849
*Themisto libellula*	Spring (PS92)	15.3–40.2 mm	WM (mg)	DM (mg)	38	0.166	− 2.869	0.934

TL = total length, SL = standard length, WM = wet mass and DM = dry mass. Seasons refer to austral seasons in case of Antarctic species

The TL–WM relationship of the 21 T*. macrura* collected during austral winter (PS81) proved robust (*R*^*2*^ > 0.98) in spite of the relatively low sample size (Table 3). DM was measured on individuals of *E. crystallorophias* collected during austral summer (PS82) and for Arctic krill species collected during spring (PS106/2). The regression model for *T. inermis* did not explain a large part of the data variability (*R*^*2*^ = 0.50, Table 3), whilst the regression model for *T. longicaudata* had a better fit (*R*^*2*^ = 0.87). Based on size, the individuals of *T. inermis* measured were likely all adults (Smith 1991). The slope value for *b* was higher for *T. longicaudata*, probably because the size range encompassed immature post-larval and adult individuals (Lindley 1978).

#### Fish

TL, WM and DM were measured for the Antarctic species *E. antarctica* and *B. antarcticus* collected during austral summer (Table 3) and TL–SL/WM–DM relationships were established (Table 4). The TL–WM/DM regression models for *E. antarctica* violated linearity assumptions. For the Arctic fish species *B. saida*, TL and WM measurements were performed on individuals caught in the under-ice surface during late summer/autumn 2012 (PS80) and at the shelf bottom during spring 2017 (PS106/2). The majority of the fish from PS80 had a TL ranging from 52 to 94 mm (David et al. 2016), likely representing 0- to 1-year-old fish (Lønne and Gulliksen 1989; Ponomarenko 2000; Laurel et al. 2017). A length–mass regression model using only the individuals in this size range did not differ significantly from a regression model including the 11 individuals with a TL > 100 mm, and all measurements were, therefore, included in the regression model (Table 3). The fish from PS106/2 were of a larger size class (86–173 mm), likely representing 1- to 2-year-old fish (Lønne and Gulliksen 1989).

#### Amphipods

Amphipod regression models were established for one Southern Ocean species and several Arctic species (Table 3). A TL–WM regression was established for *A. glacialis* caught during spring 2015 (PS92) using individuals with a TL between 7.6 and 11.1 mm (Table 3). Based on a length–frequency distribution (unpublished data), this range likely represents a single cohort or age class. DM was measured on individuals from spring (PS106/2; Table 3). The WM and DM of *T. libellula* and *Themisto abyssorum* were measured using data from several expeditions (Table 3). The TL–DM regression model for *T. abyssorum* sampled during PS106/2 violated linearity assumptions.

Summer/autumn measurements on individuals of the Arctic *Eusirus holmii* and *Onisimus glacialis* (PS80) and the Antarctic *E. laticarpus* (PS89) showed a relatively high variability in WM per TL, resulting in regression models that explained a relatively small proportion of the data (*R*^*2*^ = 0.47, 0.66 and 0.69, respectively, Table 3). Similar results were found for TL–DM models for the Arctic species (Table 3). The TL–WM model for *Onisimus nanseni* had a better fit (*R*^*2*^ = 0.96), although the number of measured individuals was relatively low (*n* = 11, Table 3).

#### Other species

**Fig. 3:**
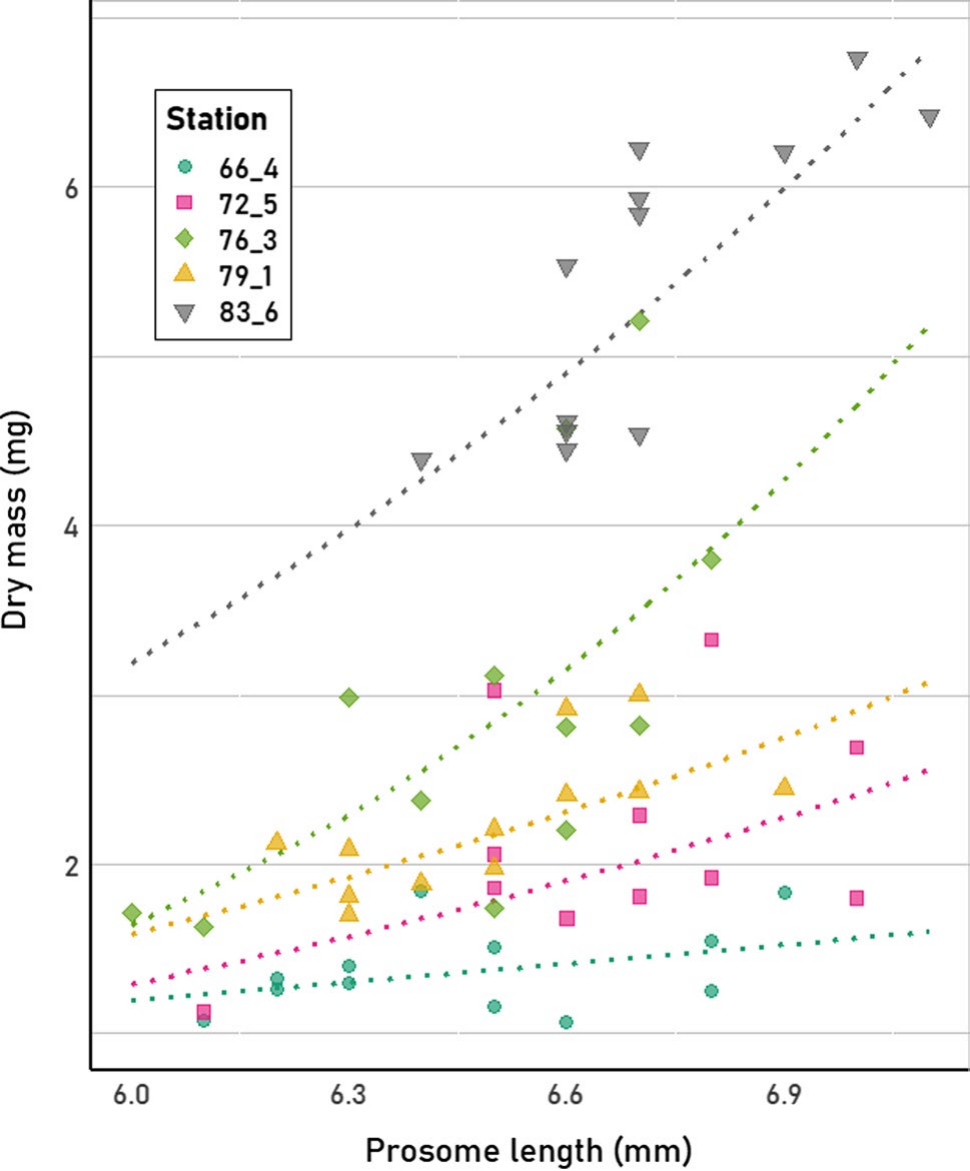
Total length–dry mass relationships of adult females of the copepod *Calanus hyperboreus* caught during summer. Although individuals from 14 stations were measured, the measurements from 5 stations are shown here to illustrate the variability in dry mass per length at different locations. All measurements can be found in Online Resource 2

The TL and DM were measured for a number of adult females of the Arctic copepod *Calanus hyperboreus*. Interestingly, the DM per length showed very high variation depending on sampling location or associated timing of sampling (Fig. 3). TL–WM and TL–DM regressions are given for the chaetognath species *Eukrohnia hamata* collected in Arctic spring (PS92, Table 3). Lastly, WM–DM relationships are given for two species of scyphozoa (*Atolla* sp. and *Periphylla periphylla*), which were collected in the Weddell Sea in austral summer (PS89, Table 4).

### Seasonal comparison of total length–mass regressions within species

#### Fish

**Fig. 4:**
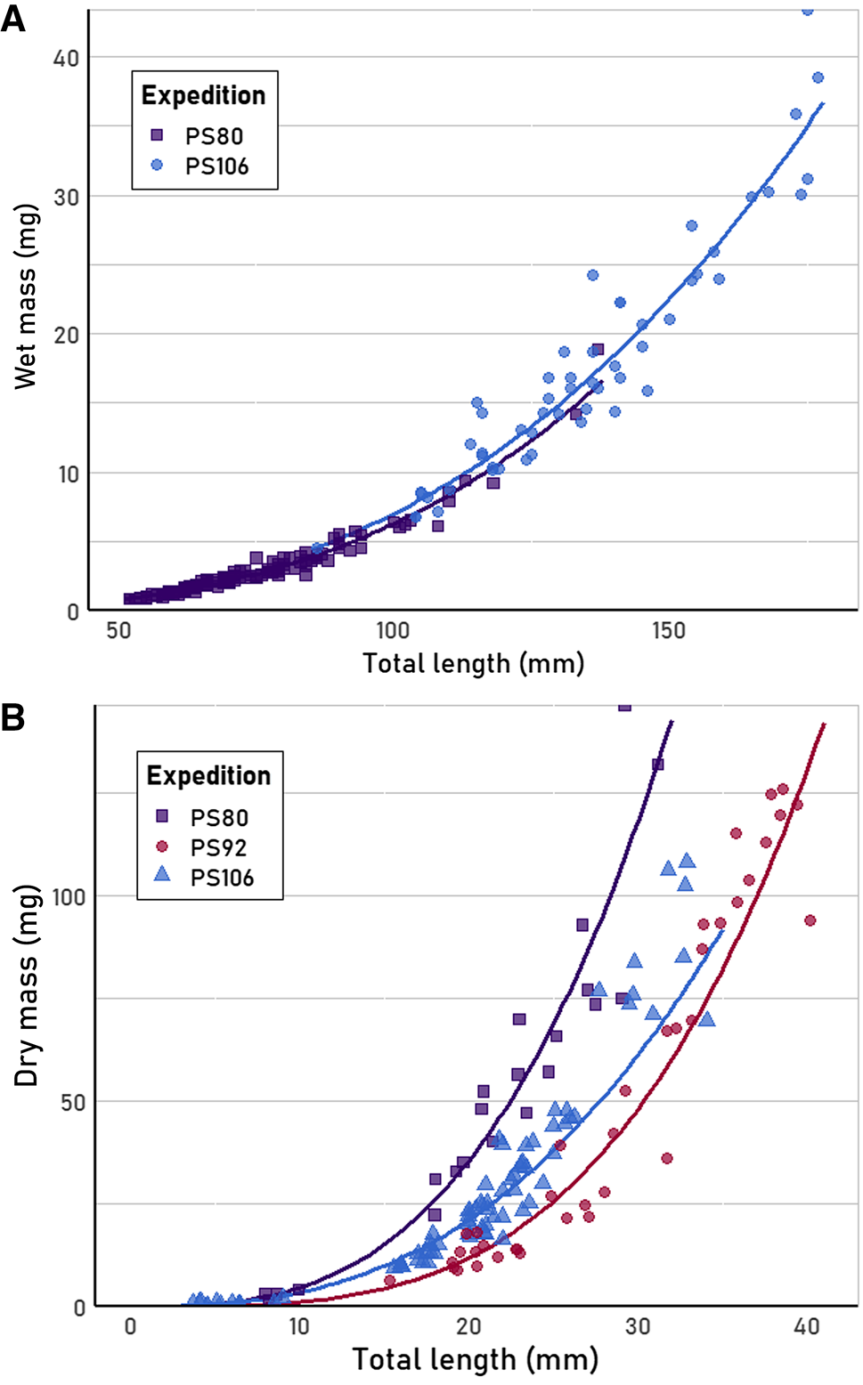
Comparison of total length – mass relationships between different seasons for **A** *Boreogadus saida* and **B** *Themisto libellula.* PS92 = May–June (2015), PS106/2 = June–July (2017), PS80 = August–October (2012)

TL–WM regression models were compared between seasons for *B. saida*. The regression models (Fig. 4A) had the same slope, but had significantly different intercepts (*F*_1, 170_ = 6.03, *p* = 0.015), suggesting that the difference in log_10_-transformed mass was constant and did not vary as a function of log_10_-transformed length between years or expeditions. A single linear regression model between TL and SL is given for all fish from all expeditions (Table 4), as there was no significant difference between models when separated by expedition.

#### Amphipods

Comparing the TL–WM regression of *T. libellula* from PS80 (summer) with that of individuals measured from PS92 (spring), a significant difference in both slopes (*F*_1, 114_ = 11.17, *p* = 0.001) and intercepts (*F*_1, 115_ = 120.95, *p* < 0.0001) was found. This was also the case for the TL–DM regression models of PS80 and PS92 (slope: *F*_1, 78_ = 6.09, *p* = 0.016, intercept: *F*_1, 79_ = 191.62, *p* < 0.0001; Table 3, Fig. 4B). DM of individuals collected later in spring (PS106/2) were also measured (Fig. 4B). The resulting model differed significantly in intercept compared to the models of PS80 (*F*_1, 40_ = 82.28, *p* < 0.0001) and PS92 (*F*_1, 78_ = 43.90, *p* < 0.0001).

When comparing TL–DM regressions for *T. abyssorum* caught during two spring expeditions (PS92 and PS106) results showed similar findings. The regression models showed a significant difference in slope (*F*_1, 47_ = 7.188, *p* = 0.01). The model for individuals from PS106/2, however, violated the linearity assumption suggesting that a power function may not adequately represent the data.

### Comparison of regressions separated by developmental stage, sex or age

#### Krill

**Fig. 5:**
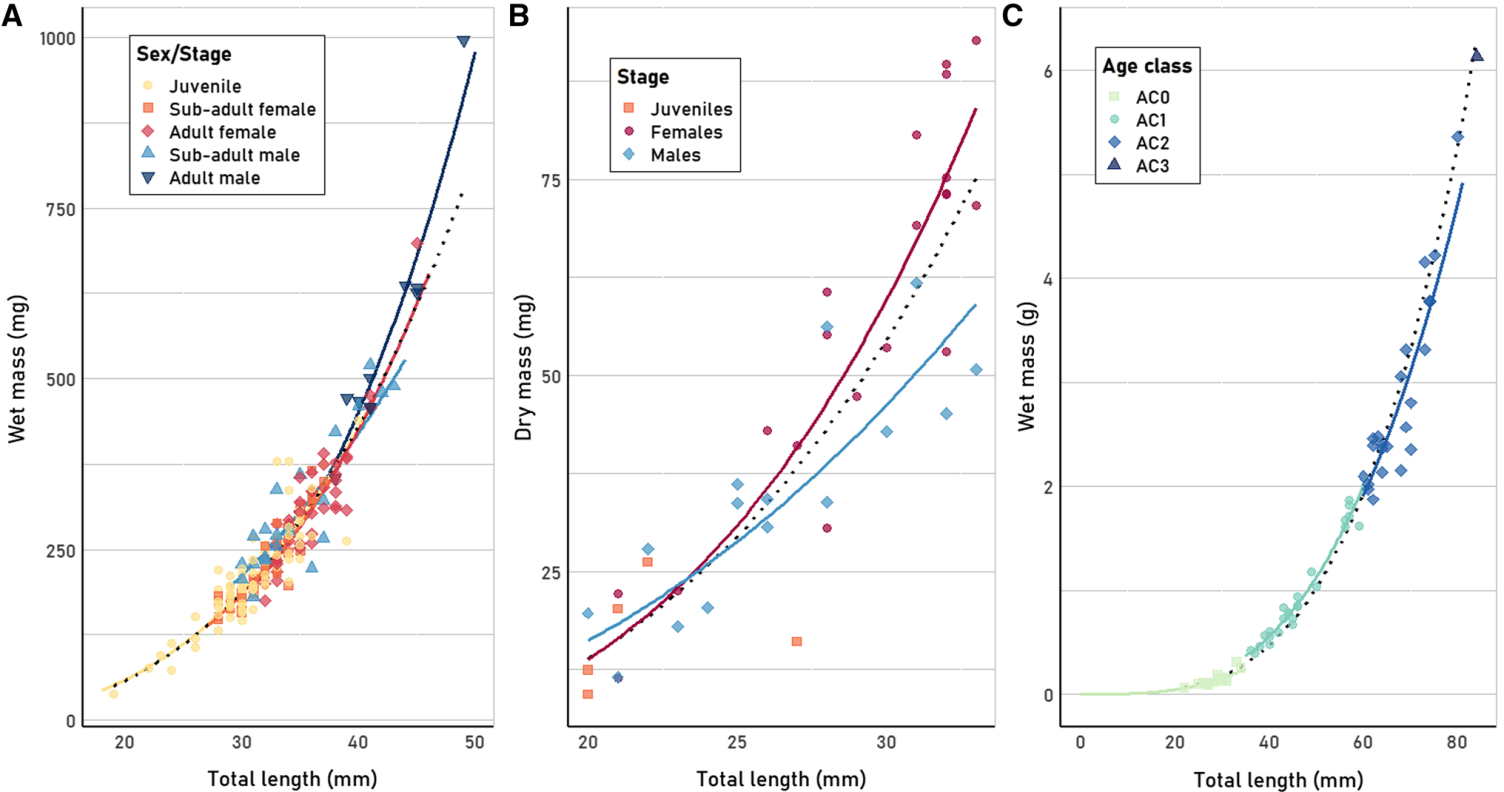
Comparison of total length–mass relationships between different sexes/developmental stages or age classes of **A** *Euphausia superba* caught during summer 2014/2015 (PS89), **B** *Euphausia crystallorophias* caught during summer 2013/2014 (PS82) and **C** *Electrona antarctica* caught during summer 2014/2015 (PS89). Regression models using all data combined are indicated with dotted lines

The 430 measurements on Antarctic krill from PS89 allowed for a further analysis of regression models from different developmental stages and sexes (Table 5). Individuals of Antarctic krill from austral summer (PS89) were divided in the developmental stages juveniles, sub-adult females, adult females (not gravid), sub-adults males and adult males (Fig. 5A). The TL–WM regression models were not significantly different between sub-adult and adult females, nor between sub-adult and adult males. Comparing males and females (using both sub-adults and adults), the regression models did not show any difference in slope, although they differed in intercept (*F*_1, 112_ = 11.11, *p* = 0.001), suggesting that the average difference in log_10_-transformed mass between males and females is constant and does not vary as a function of log_10_-transformed length. In the regression model for juveniles, no significant difference was found when compared to either females or males. None of the sex/stage-specific regressions models for *E. superba* differed significantly from the model using all austral summer measurements. The regression model subsets did also not consistently decrease the residual standard error compared to the model using the complete austral summer dataset (Table 5). Comparing the TL–WM regression for austral winter (PS81) furcilia and juvenile krill with austral summer (PS89) juvenile krill, no significant difference was found between slopes, but there was a significant difference between intercepts (*F*_1, 206_ = 7.07, *p* = 0.008).

**Table 5:** Comparison between length–mass regression models of different developmental stages or ages of the Southern Ocean species *Euphausia superba* (austral summer PS89), *Euphausia crystallorophias* (austral summer PS82) and *Electrona antarctica* (austral summer PS89) and the Arctic Ocean species *Themisto libellula* (summer PS80)

Species	Stage	Length range (mm)	*x*	*y*	*n*	log *a*	*a*	*b*	*R*^*2*^	*df*	Res. St. error
*Euphausia superba*	All	19–49	TL (mm)	WM (mg)	215	− 2.011	0.010	2.900	0.889	213	0.057
*Euphausia superba*	Juveniles	19–40	TL (mm)	WM (mg)	100	− 1.966	0.011	2.870	0.837	98	0.061
*Euphausia superba*	Sub-adult female	28–37	TL (mm)	WM (mg)	44	− 1.946	0.011	2.851	0.834	42	0.042
*Euphausia superba*	Adult females	32–45	TL (mm)	WM (mg)	40	− 2.284	0.005	3.067	0.809	38	0.050
*Euphausia superba*	Sub-adult males	30–43	TL (mm)	WM (mg)	22	− 1.206	0.062	2.390	0.757	20	0.067
*Euphausia superba*	Adult males	38–49	TL (mm)	WM (mg)	9	− 2.905	0.001	3.470	0.933	7	0.036
*Euphausia crystallorophias*	All	20–33	TL (mm)	DM (mg)	40	− 3.246	0.001	3.372	0.803	38	0.124
*Euphausia crystallorophias*	Female	21–33	TL (mm)	DM (mg)	20	− 3.559	0.000	3.611	0.860	18	0.092
*Euphausia crystallorophias*	Male	20–33	TL (mm)	DM (mg)	15	− 2.162	0.007	2.590	0.731	13	0.109
*Electrona antarctica*	All^c^	22–84	TL (mm)	WM (mg)	68	− 5.924	0.000	3.494	0.983	66	0.072
*Electrona antarctica*	AC0	22–34	TL (mm)	WM (mg)	19	− 5.379	0.000	3.093	0.750	17	0.080
*Electrona antarctica*	AC1	36–59	TL (mm)	WM (mg)	25	− 5.331	0.000	3.168	0.964	23	0.041
*Electrona antarctica*	AC2	60–80	TL (mm)	WM (mg)	23	− 5.329	0.000	3.155	0.808	21	0.055
*Electrona antarctica*	All^c^	22–84	TL (mm)	DM (mg)	47	− 7.397	0.000	4.022	0.968	45	0.071
*Electrona antarctica*	AC0	22–34	TL (mm)	DM (mg)	18	− 5.725	0.000	2.848	0.432	16	0.149
*Electrona antarctica*	AC1	37–59	TL (mm)	DM (mg)	13	− 6.120	0.000	3.303	0.924	11	0.067
*Electrona antarctica*	AC2	60–75	TL (mm)	DM (mg)	15	− 6.267	0.000	3.391	0.623	13	0.084
*Themisto libellula*	All	6–28	TL (mm)	WM (mg)	58	− 1.595	0.025	2.893	0.988	56	0.071
*Themisto libellula*	Immature/mature	18–28	TL (mm)	WM (mg)	27	− 0.806	0.156	2.300	0.765	25	0.062
*Themisto libellula*	Juveniles	6–11	TL (mm)	WM (mg)	31	− 1.444	0.036	2.722	0.836	29	0.074

AC = age class, TL = total length, WM = wet mass, DM = dry mass

^c^Data violates linearity assumption

The separate TL–DW regression models for males and females of *E. crystallorophias* did not differ significantly from each other or from a model using all available measurements (Table 5; Fig. 5B), including a small number of juveniles (*n* = 5). The regression model subsets by sex did, however, decrease the residual standard error compared to the model using the complete dataset (Table 5).

#### Fish

For *E. antarctica*, TL–DW/WW regression models were compared between ages (Fig. 5C). No statistical differences were found between the slopes of the regression using all data and the slopes of the regressions separated per age class (Table 5). Only the intercept of the regression model using age-class 1 data differed significantly from the model using all data combined (TL-WM: *F*_1, 90_ = 11.71, *p* = 0.001; TL–DM: *F*_1, 56_ = 5.19, *p* < 0.03). Comparing the regression models per age class with each other, there were also no significant differences between slopes, but one significant difference between the intercepts of age classes 0 and 1 (*F*_1, 41_ = 18.6, *p* < 0.0001). Data plots showed that the measurements from age-class 1 were all located above the regression line established using all data (Fig. 5C).

#### Amphipods

During summer/autumn 2012 (PS80) two size classes of *T. libellula* were found which could be attributed to different developmental stages. Based on sex and stage determination of several individuals from this expedition and information on the development of *T. libellula* in the studies of Percy (1993), Koszteyn et al. (1995) and Auel and Werner (2003), the amphipods smaller than 12 mm were defined as juveniles and the individuals > 15 mm were regarded as a separate age group consisting of immature and mature individuals. The TL–WM relationship of *T. libellula* from PS80 was not significantly different between males (*n* = 9) and females (*n* = 17) or juveniles and immature/mature individuals (Table 5).

### Other allometric relationships

Austral winter (PS81) relationships between CL, TL and WM are given for age-class 0 (furcilia and juveniles) Antarctic krill < 24 mm (Table 6). The relationship between CL, TL and WM of *T. macrura* collected in winter can also be found in Table 6. CL seemed to be a good predictor for both TL and WM of both species, *R*^*2*^ values being somewhat higher for *T. macrura* (0.98 and 0.96, respectively) than for age-class 0 *E. superba* (0.86 and 0.81, respectively). Although the size range of *T. macrura* in this analysis is similar to that of age-class 0 Antarctic krill, these individuals represent older developmental stages. The size range of 9–26 mm indicates that the measured *T. macrura* encompassed both juvenile and adult individuals (Nordhausen 1994).

**Table 6:** Relationship between body parts, total length and mass of several Southern and Arctic Ocean species

Species	Season	Function	*x*	*y*	*n*	*a*	*b*	*R*^*2*^
*Apherusa glacialis*	Summer (PS80)	*y* = *ax* + *b*	TSL (mm)	TL (mm)	656	19.74	2.062	0.723
*Apherusa glacialis*	Summer (PS80)	*y* = *ax* + *b*	ELH (mm)	TL (mm)	654	16.677	1.561	0.728
*Apherusa glacialis*	Summer (PS80)	*y* = *ax* + *b*	ELV (mm)	TL (mm)	654	19.2	1.615	0.702
*Bathylagus antarcticus*	Summer (PS89)	*y* = *ax* + *b*	OL (mm)	TL (mm)	11	33.458	− 0.623	0.812
*Bathylagus antarcticus*	Summer (PS89)	*y* = *a x*^*b*^	OL (mm)	WM (g)	11	0.102	4.046	0.869
*Bathylagus antarcticus*	Summer (PS89)	*y* = *a x*^*b*^	OL (mm)	DM (g)	11	0.017	3.970	0.872
*Bathylagus antarcticus*	Summer (PS89)	*y* = *ax* + *b*	OLW(mm)	TL (mm)	11	69.545	− 7.491	0.828
*Bathylagus antarcticus*	Summer (PS89)	*y* = *a x*^*b*^	OW (mm)	WM (g)	11	1.224	4.394	0.847
*Bathylagus antarcticus*	Summer (PS89)	*y* = *a x*^*b*^	OLW(mm)	DM (g)	11	0.193	4.303	0.846
*Boreogadus saida*^*c*^	Summer (PS80)	*y* = *ax* + *b*	OL (mm)	TL (mm)	138	24.27	20.713	0.943
*Boreogadus saida*^*c*^	Summer (PS80)	*y* = *a x*^*b*^	OL (mm)	WM (g)	108	0.506	2.076	0.928
*Electrona antarctica*	Summer (PS89)	*y* = *ax* + *b*	OL (mm)	TL (mm)	68	33.767	2.556	0.965
*Electrona antarctica*	Summer (PS89)	*y* = *a x*^*b*^	OL (mm)	WM (g)	69	0.3388	3.283	0.986
*Electrona antarctica*	Summer (PS89)	*y* = *a x*^*b*^	OL (mm)	DM (g)	48	0.079	3.785	0.974
*Electrona antarctica*	Summer (PS89)	*y* = *ax* + *b*	OW (mm)	TL (mm)	68	42.89	2.074	0.973
*Electrona antarctica*	Summer (PS89)	*y* = *a x*^*b*^	OW (mm)	WM (g)	69	0.714	3.307	0.981
*Electrona antarctica*	Summer (PS89)	*y* = *a x*^*b*^	OW (mm)	DM (g)	48	0.182	3.845	0.980
*Eukrohnia hamata*^*d*^	Spring (PS92)	*y* = *ax* + *b*	HW (mm)	TL (mm)	70	15.55	2.795	0.726
*Eukrohnia hamata*^*d*^	Spring (PS92)	*y* = *ax* + *b*	TLL (mm)	TL (mm)	186	4.500	0.601	0.967
*Eukrohnia hamata*^*d*^	Spring (PS92)	*y* = *ax* + *b*	ED (mm)	TL (mm)	30	61.745	11.644	0.361
*Euphausia superba*	Winter (PS81)	*y* = *ax* + *b*	CL (mm)	TL (mm)	101	3.254	0.280	0.858
*Euphausia superba*	Winter (PS81)	*y* = *a x*^*b*^	CL (mm)	WM (mg)	101	0.187	3.057	0.811
*Eusirus laticarpus*	Summer (PS89)	*y* = *ax* + *b*	HL (mm)	TL (mm)	28	5.180	9.830	0.634
*Parasagitta elegans*	Spring (PS92)	*y* = *ax* + *b*	HW (mm)	TL (mm)	11	23.527	− 1.236	0.925
*Parasagitta elegans*	Spring (PS92)	*y* = *ax* + *b*	TLL (mm)	TL (mm)	29	5.249	0.333	0.923
*Themisto libellula*	Summer (PS80)	*y* = *ax* + *b*	TSL (mm)	TL (mm)	447	17.371	2.051	0.959
*Themisto libellula*	Summer (PS80)	*y* = *ax* + *b*	ELH (mm)	TL (mm)	401	11.115	− 3.318	0.918
*Themisto libellula*	Summer (PS80)	*y* = *ax* + *b*	ELV (mm)	TL (mm)	401	8.483	− 4.689	0.953
*Thysanoessa macrura*	Winter (PS81)	*y* = *ax* + *b*	CL (mm)	TL (mm)	21	3.101	2.794	0.977
*Thysanoessa macrura*	Winter (PS81)	*y* = *a x*^*b*^	CL (mm)	WM (mg)	21	0.464	2.685	0.962

Abbreviations as defined in Table 2. Seasons refer to austral seasons in case of Antarctic species

^c^Previously published in David et al. (2016)

^d^Previously published in Immerz (2016)

TSL, ELH and ELV were measured for the Arctic amphipods *A. glacialis* and *T. libellula* caught during late summer/autumn (PS80; Table 6). All measurements were a relatively good predictor for the TL in *T. libellula* (*R*^*2*^ = 0.89–0.95), whilst the values for the models on *A. glacialis* measurements were somewhat lower (*R*^*2*^ = 0.66–0.72). For this latter species, the regression using horizontal eye length gave the best results based on *R*^*2*^ (Table 6). The head length of *E. laticarpus* proved to be a mediocre predictor for TL due to high residual variability (*R*^*2*^ = 0.63).

From the fish species *B. antarcticus, E. antarctica* (Southern Ocean) and *B. saida* (Arctic Ocean), the otoliths were measured and related to TL, WM and DM (Table 6). The low residual variability in the regression models of all three species (*R*^*2*^ = 0.81–0.99) indicated that both OL and OW were reliable predictors of TL (Table 6).

The HW and TLL of chaetognaths were measured as a predictor of TL for the Arctic species *E. hamata* and *P. elegans* (Table 6). Particularly TLL appeared to be a good predictor of TL, whilst HW showed higher variability. ED was not a good predictor for the TL of *E. hamata* (*R*^*2*^ = 0.36).

## Discussion

There is a general lack of knowledge on length–mass relationships of important polar species or the sources of variability in mass at a given length. This study fills knowledge gaps by providing length–mass regressions of ecologically important species of Antarctic and Arctic zooplankton and nekton in the winter season, of the less studied developmental stages, such as krill furcilia and juveniles, and of species that are increasingly recognized as important parts of the food web but still lack allometric analysis, such as chaetognaths and jelly fish. The more accurately the drivers of the variability of allometric relationships of polar zooplankton and nekton can be constrained, the more reliable can they be used in studies where direct allometric measurements of animal populations are not possible. With the presented regression models for certain body parts of key species we intend to contribute to the improvement of polar food web studies and carbon flux models.

For some of the studied species, the sample size was relatively low. Different recommendations regarding minimum *n* are present in literature. A recommended sample size of *n* = 10–20 has been suggested for ecological studies (Gotelli and Ellison 2004). Jenkins and Quintana-Ascencio (2020) recommend a minimum sample size of *n* > 8 for a dataset with low variance and *n* > 25 for a dataset with high variance to find the model that adequately represents the data, indicating that *n* > 25 is sufficient in any case. Note that variance here refers to the variance in the original-sampled population and not the variance that is explained by the model (which is *R*^*2*^; Cornell & Berger 1987; Jenkins and Quintana-Ascencio 2020). A high *R*^*2*^ for a model established with a low number of samples could be a result of the natural variance not being well represented by the data (Cornell and Berger 1987). Sample size should thus be noted when using the presented regression models. In addition, it is recommended to use the regression models for the reported size ranges and developmental stages.

### The use of regression models for biomass estimation of Antarctic euphausiids

In previous studies of Antarctic euphausiids, length as a predictor of mass was found to be influenced by the differences between sexes, developmental stages, seasons and regions, for both *E. superba* and *T. macrura* (Morris et al. 1988; Siegel 1992; Färber-Lorda 1994; Schmidt et al. 2014). In these studies, length–mass regressions (both WM and DM) were found to be more accurate when models were separated by sex and stage. However, in the case of *E. superba*, comparable accuracy was found when separating the krill into the groups ‘males’, ‘gravid females’, ‘non-gravid females’ and ‘spent females’ (the latter in the post-spawning period) according to Siegel (1992) or ‘adult males’, ‘gravid females’ and ‘standard krill’ according to Morris et al. (1988). Similar findings were shown by Atkinson et al. (2006), who showed that the intercepts in the regression models for gravid females and adult males differed from the model combining all stages in summer, including juveniles. The results indicated that females were heavier than males of the same length (Atkinson et al. 2006). The regression models of krill caught in austral summer (PS89) studied here correspond with these earlier findings. When taking results of previous studies into account, our results suggest that a single model, including juveniles, sub-adult and adult females and likely sub-adult males, would be sufficient for an accurate prediction of wet mass based on total length for these stages in summer. There were only a few adult males in this study (*n* = 8) and that separating these may improve mass predictions, as was also suggested when looking at the residual standard error.

Although *E. superba* is a well-studied species and several studies report information on length and mass (overviews in Morris et al. 1988 and Siegel 1992), the majority of information is about post-larval krill. For larval and juvenile Antarctic krill there is much less information, as well as for the months outside summer. Some studies provide a total length–wet mass relationship for size ranges that include larvae and juveniles, but do not separate them from sub-adult and adult krill (e.g. Daly 1990; references in Siegel 1992). We found no statistical difference in slope between the length–mass regression of furcilia and juveniles caught in austral winter and larger juveniles caught in austral summer, but a difference in intercept between the two, suggesting there are little differences in morphological traits between and within these stages, but that the summer juveniles were consistently heavier. This is not surprising as there is not only a difference in food availability between the seasons, the summer juveniles may also be a year older (Siegel 1987).

**Fig. 6:**
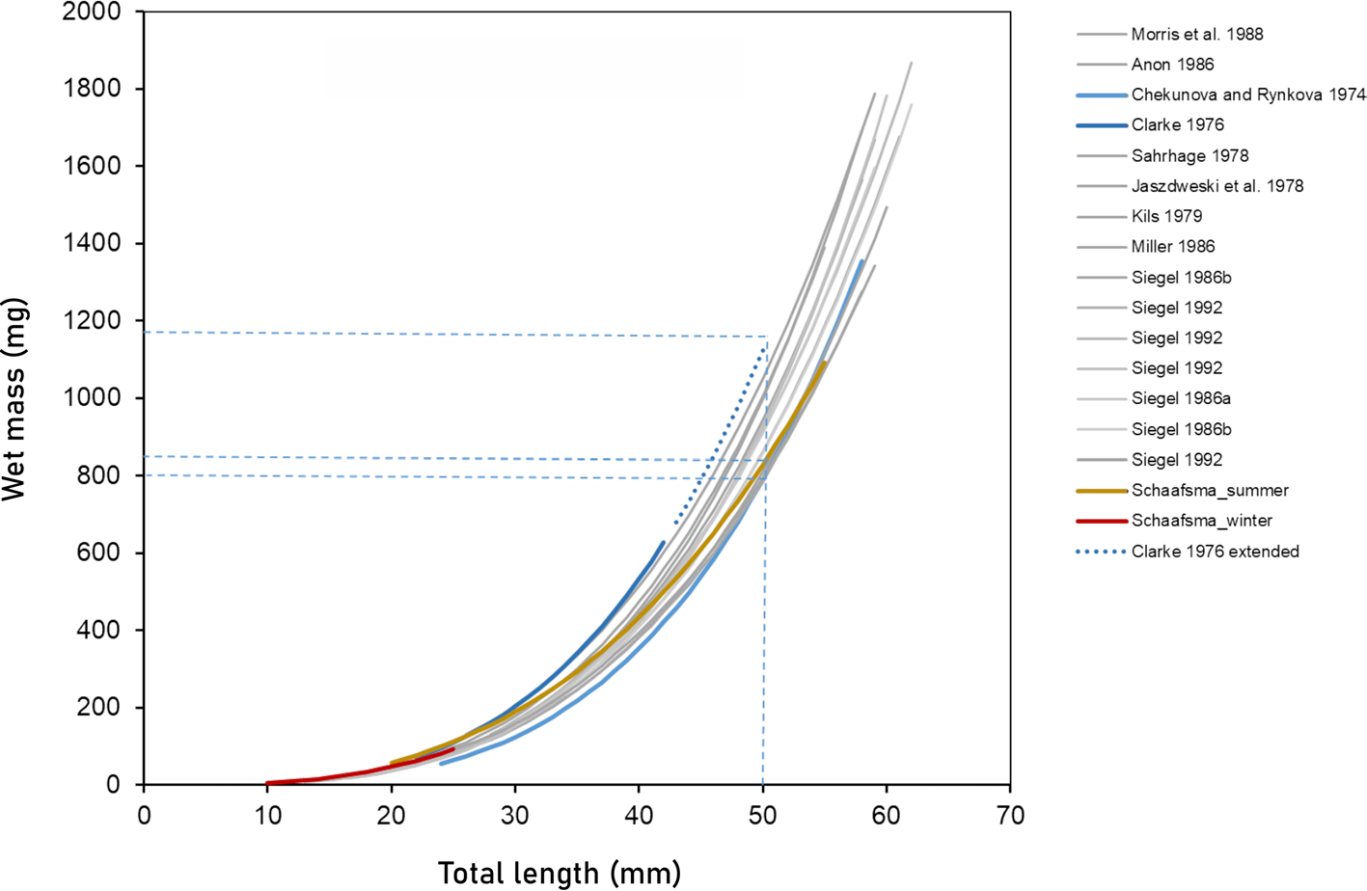
Length–mass regression models for *Euphausia superba* from various studies since 1974. The regression functions resulting in the lowest (Chekunova and Rynkova 1974) and highest (Clarke 1976) masses at lengths up to 50 mm were highlighted in blue. Orange and red regression functions depict this study’s data from austral summer (juveniles and adults) and austral winter (age-0 furcilia and juveniles), respectively. For a krill of 50-mm length, the estimated highest and lowest masses compared to the present study are indicated with light-blue-dashed lines

The relatively high number of TL–WM regression models for *E. superba* available in literature (and available through the R package “solong”; SCAR 2021) can give an indication of the effect of using any model for a biomass estimate and show that variability in regression parameters can be found (Fig. 6). When comparing these available models, results indicate that the most accurate length–mass relationships are based on direct measurements from the sampled population in any given study. Given previously discussed findings, this may largely depend on the presence or absence of gravid females and adult males. When representative sampling of the krill population is not possible, e.g. due to limited spatial coverage or non-quantitative sampling gear, literature values can be a useful means to estimate biomass from length–frequency data, especially with respect to the rapidly developing autonomous acoustic sampling devices, such as moorings and gliders (Reiss et al. 2021). The comparison of multiple published regression models of Antarctic krill indicates that the maximum overestimate of the biomass of krill of 50 mm TL could be close to 50%, whereas the maximum underestimate would range in the order of 30% (Fig. 6). The uncertainty of these length–mass models should be considered in hydroacoustic biomass estimates, when direct sampling of krill is not possible or not appropriate, for example, when size-selective nets are used to sample the krill population. Results indicate that, firstly, it is valuable to have a large number of regression models available in either literature or public databases, to be able to use the ones that closely resembles the season, region and size structure of the investigated population for the most accurate estimate of biomass and, secondly, information on timing of life cycle events and maturation in a certain region and season is very useful for the selection of the model best representing the encountered population.

To our knowledge, the present study is the first to present austral winter length–mass regression models for *T. macrura*. The individuals of *T. macrura* were not staged, so a comparison between stages/sexes could not be conducted. A previous summer study found that the length–mass regressions for sub-adults and adults were not significantly different from each other, although significant differences were found when males and females were separated (Färber-Lorda 1994). For *E. superba*, however, no significant differences were found between the length–mass relationship of males and females during the winter resting stages (Siegel 1986a), and general length–mass relationships are deemed sufficient for the pre-spawning and winter periods (Siegel 1992). Because the *T. macrura* in the current study were also collected in the winter season and growth rate is suggested to decrease during the winter months for this species (Haraldsson and Siegel 2014), a general regression model may be sufficient for estimating mass per length of this species, as suggested for *E. superba* (Siegel 1992). However, the spawning season of *T. macrura* may start early in the year. Depending on region (Makarov 1979; Everson 2000), it may potentially be initiated as early as mid-winter (Haraldsson and Siegel 2014). This indicates that information on the presence of gravid females in a population and knowledge on the length–weight relationships of different developmental stages and regions during winter is necessary to fully understand possible sources of variation in regression models. Many aspects of the reproductive cycle and life history traits of *T. macrura* are still poorly understood (Wallis et al. 2018).

Austral summer TL–DM relationships of *E. crystallorophias* did not statistically differ between sexes. Despite the relatively low sample sizes, this suggests that a single regression model can be used to establish DM. However, not all developmental stages were represented and it is likely that gravid and spent females would warrant a separate model similar to *E. superba*. Further TL–WM relationships for individuals caught in austral summer, with juveniles, males and females separated, can be found in Pakhomov and Perissinotto (1996), but the reported regression models were not analysed for statistical differences.

### Separating age classes of *Electrona antarctica*

Different length–mass relationships are often found for different age classes and life stages of fish (Froese 2006 and references therein). Younger individuals grow relatively more in length than in body mass, compared to older individuals, as they invest more energy in becoming larger (Fulton 1904; Froese 2006; Van de Putte et al. 2006; Schaafsma et al. 2018). In addition, seasonal and annual variability can be found and it is generally recommended that differences between sexes are tested (Froese 2006). The results of comparing the regression models of different age classes of *E. antarctica* remain inconclusive about using a general model for all, as age-class 1 seemed to warrant a separate regression model, whilst age classes 0 and 2 did not. This may be attributed to the age classification according to Greely et al. (1999), which were based on a relatively low number of samples. In addition, *E. antarctica* lacks distinct annual rings in their otoliths, hampering age estimation (Linkowski 1987; Rowedder 1979; Greely et al. 1999). Furthermore, the model using all available data violated model assumptions. Relatively large amounts of data are available on length–mass relationships of fish from the Southern Ocean (e.g. Gon and Heemstra 1990; Pakhomov et al. 1996; Eastman and DeVries 2000; Casaux et al. 2003; Kock and Jones 2005; Reid et al. 2007; Flores et al. 2008; Wei et al. 2017; Escobar-Flores et al. 2020; Saunders et al. 2020), but some of these analyses include fish sampled over several seasons and often no differentiation is made between sexes, developmental stages or age classes. The latter maybe a consequence of the difficulty in age estimation.

### Influence of size, sex, season, region or habitat?

Most polar cod (*B. saida*), caught during the end of Arctic summer/autumn (PS80), probably belonged to the same year class and only five of the measured fish were females. Therefore, a comparison between age classes or sexes was not possible. Fey and Węsławski (2017) did not find any difference between length–wet mass regressions of males and females, analysed on individuals from 61 to 240 mm collected in the Svalbard fjords in September/October. The fish from PS80 and PS92 were caught in the under-ice surface waters over the deep basin of the Central Arctic Ocean. Most fish in previous studies, as well as fish caught during the other expedition in this study (PS106/2), were caught in shallow coastal waters (Frost and Lowry 1981; Finley et al 1990; Nahrgang et al. 2014; Koenker et al. 2018; Copeman et al. 2020). It is hypothesized that young polar cod descend to deeper water layers or remain in the surface waters depending on timing of hatching (Geoffroy et al. 2016). Hatching time may thus have consequences for length–weight regression parameters that may represent variation in the growth and dynamics of populations with different life cycles, occupying these different habitats. A comparison between the polar cod from the two expeditions in this study is, however, unable to give an indication if resulting differences are due to age classes, season, sampling location or habitat. Also when compared to length–mass relationships from aforementioned references, no clear effect of sampling habitat on length–mass relationships can be determined. There are indications that there is variation depending on sampling location although this may also be seasonal (Geoffroy et al. 2016 and references therein). Dupont et al. (2020) suggested that winter sea-ice concentration affects the period suitable for growth of polar cod, indicating that environmental conditions could indeed influence the length–mass relationship of certain age classes.

In earlier studies, the relationship between TL and WM was found to differ between males and females for the amphipods *A. glacialis* and *Gammarus wilkitzkii* (Poltermann 2000). This was not the case for *T. libelulla* in this study, which showed no differences between regression models of both sexes, although the sample size was quite low. The models suggested an influence of season on WM and DM, which increased from spring to end of summer for *T. libellula* (Fig. 4B). Despite one of the models violating linear regression assumptions, similar findings can be suggested for *T. abyssorum*. A variability in TL–DM relationships between seasons, with lower dry masses for given total lengths in winter compared to summer, was previously also found for *A. glacialis* and *G. wilkitzkii*, but not for *Onisimus* spp., although measured animals did not always cover the same length range in both seasons (Werner and Auel 2005).

Individuals of *T. libellula* were separated in two age classes according to information from several studies. In other studies, however, the species was found to mature at larger sizes. *T. libellula* was found to mature at approximately 19–21 mm, with a maximum TL of about 25 mm, at south-eastern Alaska (Wing 1976), whilst it was found to mature when exceeding 35 mm, with a maximum TL of approximately 46 mm at Baffin Island, Canadian Arctic (Dunbar, 1946). These differences, including the different life cycles of both populations, were attributed to the latitudinal gradient in water temperature (Wing 1976). This suggests large differences in growth and maturation between regions in addition to seasons.

A high variability in the DM of *C. hyperboreus* like in our study was found before in several developmental stages (Hirche 1997; Ashjian et al. 2003). In our data from PS106/2, this variability could partially be attributed to an increase in mass over time during the expedition, i.e. seasonal progression. Sampling was, however, performed within two weeks. Therefore, differences in the extent of the mass increase with an increase in length (slope) between stations, as well as the high variability in mass per given length within a station, might be better explained by varying local food availability. For example, *C. hyperboreus* is known to depend on ice algae at least during parts of its life cycle (Kohlbach et al. 2016). In addition, females of *C. hyperboreus* store reserves for overwintering and reproduction by extensive feeding during summer so that their spawning during winter/early spring is fuelled by internal lipid reserves (Conover 1988; Falk-Petersen et al. 2008; Kosobokova and Hirche 2009).

For chaetognaths there is also likely a large seasonal difference in length–mass regression models, as a model from autumn (Richter 1994) showed a much lower value for exponent *b* (0.165) than the regression models established in this study. Chaetognaths are known to feed year-round, but studies have indicated that the feeding rate, and also growth, is lower in the winter months compared to spring and summer (Grigor et al. 2014, 2015).

### Other predictors of total length or mass

In diet studies on krill predators, using stomach contents or scat analysis, whole krill are rarely available. In these cases, CL can be the best available parameter to estimate the size of ingested krill (Hill 1990). In this study, we present a CL–WM relationship for age-class 0 furcilia and juvenile *E. superba*, indicating that CL is a reliable predictor of TL and WM in these young krill. Most previous studies focused on older krill and suggest that the CL of *E. superba* is a poor predictor of TL and WM when sexes and stages are not separated (e.g. Siegel 1982; Morris et al. 1988; Hill 1990). Therefore, it should be considered that when CL is used to estimate reconstructed TL or WM, it is not very reliable for growth studies or comparative studies of krill populations in the absence of information on sex and maturity (Morris et al. 1988; Färber-Lorda 1990, 1994). In our study, the relationship between CL and TL or WM of *T. macrura* appeared to be robust enough to be used in size and mass reconstructions of diet studies. However, in a previous summer study the CL–TL relationship was found to differ between juvenile, adult male and adult female individuals (Färber-Lorda 1990). In another study, the CL–WM relationship differed significantly between sub-adult and adult individuals, but no difference was found between males and females, indicating that morphological differences as a function of WM seem to be less pronounced compared to differences as a function of size (Färber-Lorda 1994).

Otoliths are very useful for the identification of fish species in the food or scats of their predators, as well as in archaeological and prehistoric samples. Previous investigations of the relationship between OL and fish TL indicated that the deviation between measured fish length and estimated fish length using regressions is very small and that otoliths are thus an excellent predictor for length (Frost and Lowry 1981). Saunders et al. (2020) found that OW was a slightly better predictor of SL that OL based on *R*^2^, which is consistent with our findings for *E. antarctica* and *B. antarcticus* using TL. For mass estimates, however, one (OL or OW) was not consistently better than the other, but both were robust. A lot of work has been done on otoliths of Southern Ocean fishes and many regression models are available in literature (overviews in, e.g. Hecht 1987; Gon and Heemstra 1990; Reid 1996; Saunders et al. 2020). Also for the Arctic species *B. saida*, several studies exist that relate otoliths to total fish length and mass (Frost and Lowry 1981; Finley et al. 1990; Harvey et al. 2000; Fey and Węsławski 2017). Direct comparisons between published relationships may, however, be difficult due to the use of different measures for fish length (such as total length, standard length or fork length).

Telson and eye length seem to be good predictors for TL for *T. libellula*. This is very useful for predicting biomass in the stomach contents of predators, as, again, complete bodies are often absent. *Themisto libellula* has often been reported as an important prey item for fish, such as polar cod (e.g. Lønne and Gulliksen 1989; Majewski et al. 2016; Eriksen et al. 2020), cod and capelin (Dalpadado et al. 2001), for birds, such as thick-billed murres (*Uria lomvia*) and black-legged kittiwakes (Bradstreet and Cross 1982; Karnovsky et al. 2008) and for harp seals (Haug et al. 2021). The regression models established for these parameters using data from *A. glacialis* explained less of the variability in the data. The same was true for the HL-TL regression model for *E. laticarpus*. These models are still useful for biomass estimations in diet studies where individual prey items cannot be weighed separately, especially when different size ranges of a prey item are present in the stomach of the investigated predator. Calculating the reconstructed biomasses using regression models would provide an additional measure of prey importance enabling the investigation of the contribution of different sized prey to the diet, whilst the use of only prey counts would overemphasize the importance of small prey in large numbers (Hyslop 1980).

Chaetognaths may form a large part of the zooplankton biomass and are often the most abundant zooplankton predators, in both the Arctic and the Southern Oceans (Pakhomov et al. 1999; Kosobokova and Hirche 2000; Auel and Hagen 2002; Hopcroft et al. 2005; Flores et al. 2014; David et al. 2017; Ehrlich et al. 2020). In addition, they are prey for many species of fish in both polar regions (Lønne and Gulliksen 1989; Atkinson and Percy 1992; La Mesa et al. 2004 and references therein; Walkusz et al. 2011) and are recorded in the diets of Arctic bird species (Hartley and Fisher 1936; Lønne and Gabrielsen 1992). This makes them an important part of the food web (Pakhomov et al. 1999; Giesecke and Gonlález 2012), warranting the need for accurate biomass estimates. According to our results, regressions models can be useful for reconstructing chaetognath length in diet studies. Although TLL seems to be a better predictor for TL compared to, e.g. HW, it is likely more common to find heads in the contents of a stomach than intact tails.

### Conclusion

The large variability of length–mass regression models highlights the importance to appropriately sample Antarctic krill populations in biomass surveys and to use the best available data when direct sampling of krill is not possible, preferably with knowledge on the developmental stages that are likely present in the area and/or season. A single regression model seems to be appropriate for *E. crystallorophias* in austral summer, but a potential effect of the presence of gravid and spent females should be further investigated, which is also the case for *T. macrura* in austral winter. The length–mass relationships of the predatory amphipods *T. libellula* and *T. abyssorum*, and the copepod *C. hyperboreus* were highly sensitive for the timing of their sampling. For amphipods we recommend using a model at least from a similar season and size range when estimating biomass based on length measurements The results of the comparison between the length–mass relationships of different age classes of *E. antarctica* were inconclusive and further investigation is necessary to establish if a single regression model can be used to estimate mass from length measurements on different age classes. Our findings suggest that it would be advisable to use separate regression models for different groups of *B. saida*. The source(s) of variability (age, season, region and/or habitat) remains unclear and should be further investigated. Otoliths (fish), tail length (chaetognaths) and, taking discussed caveats into account, carapace length (krill) all seem good predictors for the total length of the investigated animals. Head width is a reasonable to good predictor for chaetognath total length, depending on species. Telson length and eye length (both horizontal and vertical) proved to be reasonable to good predictors for total length for the amphipods *A. glacialis* and *T. libellula*, respectively. We emphasize the importance of publication of data and regression models of allometric measurements.

## Supplementary Information

Below is the link to the electronic supplementary material.

Supplementary file1 (XLSX 186 kb)

Supplementary file2 (XLSX 679 kb)

Supplementary file3 (XLSX 23 kb)

## Data Availability

All data are available in the supplementary material and are stored in the publicly accessible databases PANGAEA (Schaafsma et al. 2021) and the Netherlands Polar Data Center (NPDC) under project 866.13.009 (*The imperiled role of sea ice in supporting the living resources of the polar oceans (Iceflux-NL)*). The data will also be distributed in other relevant data portals, such as the Ocean Biodiversity information System (OBIS), global biodiversity Information Facility (GBIF) and the SCAR Southern Ocean Diet and Energetics Database (SO-diet; 2021).
